# Degradation of Transcriptional Repressor ATF4 during Long-Term Synaptic Plasticity

**DOI:** 10.3390/ijms21228543

**Published:** 2020-11-12

**Authors:** Spencer G. Smith, Kathryn A. Haynes, Ashok N. Hegde

**Affiliations:** Department of Biological and Environmental Sciences, Georgia College and State University, Milledgeville, GA 31061, USA; spencer.smith1@bobcats.gcsu.edu (S.G.S.); katyahaynes@gmail.com (K.A.H.)

**Keywords:** long-term potentiation, ubiquitin, proteasome, gene expression

## Abstract

Maintenance of long-term synaptic plasticity requires gene expression mediated by cAMP-responsive element binding protein (CREB). Gene expression driven by CREB can commence only if the inhibition by a transcriptional repressor activating transcription factor 4 (ATF4; also known as CREB2) is relieved. Previous research showed that the removal of ATF4 occurs through ubiquitin-proteasome-mediated proteolysis. Using chemically induced hippocampal long-term potentiation (cLTP) as a model system, we investigate the mechanisms that control ATF4 degradation. We observed that ATF4 phosphorylated at serine-219 increases upon induction of cLTP and decreases about 30 min thereafter. Proteasome inhibitor β-lactone prevents the decrease in ATF4. We found that the phosphorylation of ATF4 is mediated by cAMP-dependent protein kinase. Our initial experiments towards the identification of the ligase that mediates ubiquitination of ATF4 revealed a possible role for β-transducin repeat containing protein (β-TrCP). Regulation of ATF4 degradation is likely to be a mechanism for determining the threshold for gene expression underlying maintenance of long-term synaptic plasticity and by extension, long-term memory.

## 1. Introduction

The ability of the nervous system to change the synaptic strength is called synaptic plasticity which allows it to store information for varying durations of time. Short-term synaptic plasticity, which underlies short-term memory, depends on the posttranslational modification of existing proteins in neurons [1]. Long-term synaptic plasticity, which forms the basis of long-term memory, relies upon the translation of pre-existing mRNAs at synaptic sites, such as dendrites, the transcription of new genes in the nucleus and the translation of newly transcribed mRNAs in the cytoplasm of neurons [2,3]. Although several transcription factors play a role in the gene expression necessary for maintaining long-term synaptic plasticity [4], a transcription factor called cAMP-responsive element binding protein (CREB) has a key function in the hippocampus, a brain region critical for encoding new long-term memories [5,6]. Gene expression by CREB is activated through phosphorylation by cAMP-dependent protein kinase (PKA) and is negatively regulated by repressor proteins [7]. A protein with a vital function in inhibiting CREB-mediated gene expression is activating transcription factor 4 (ATF4; also known as CREB2) which despite its name is a repressor [8]. CREB can induce the transcription of genes critical for long-term synaptic plasticity, only if the repression by ATF4 is relieved [8,9].

How is ATF4-mediated repression of CREB removed? Previous research showed that ATF4 is degraded by the ubiquitin-proteasome pathway (UPP) during mammalian hippocampal late phase long-term potentiation [10], a widely used model system for studying long-term synaptic plasticity. In the UPP, the substrate proteins are marked for degradation by covalent linkage to a small protein called ubiquitin to a lysine residue. To an internal lysine in the first ubiquitin another ubiquitin is attached and by successive additions of several ubiquitin molecules a polyubiquitin chain is formed. The polyubiquitinated substrate is then targeted to a proteolytic complex called the proteasome for degradation [11,12]. Beyond regulating the quantities of transcription repressors such as ATF4, the UPP has wide-ranging roles in long-term synaptic plasticity and memory [13].

In the present study, we investigated the mechanisms that regulate degradation of ATF4 using chemically induced long-term potentiation (cLTP) [14] in the hippocampus as a model system because it facilitates molecular studies by modifying the bulk of the synapses in hippocampal slices and it has been shown through rigorous electrophysiological studies that the cLTP protocol induces LTP that is comparable to electrically induced LTP [10]. Specifically, based on the hypothesis that phosphorylation of ATF4 at a specific serine residue (Ser-219; pSer219-ATF4) is critical for its degradation, we followed the fate of phospho-ATF4 using immunohistochemistry and confocal microscopy on slices fixed after cLTP under various conditions. We obtained evidence for proteolysis of pSer219-ATF4 by the proteasome and that PKA is responsible for its phosphorylation. In addition, we report our initial results toward identification of the ligase that attaches ubiquitin to ATF4 to target it for proteasome-mediated degradation.

## 2. Results

### 2.1. Degradation of Phosphorylated ATF4 and its Stabilization by a Proteasome Inhibitor during cLTP

**Figure 1:**
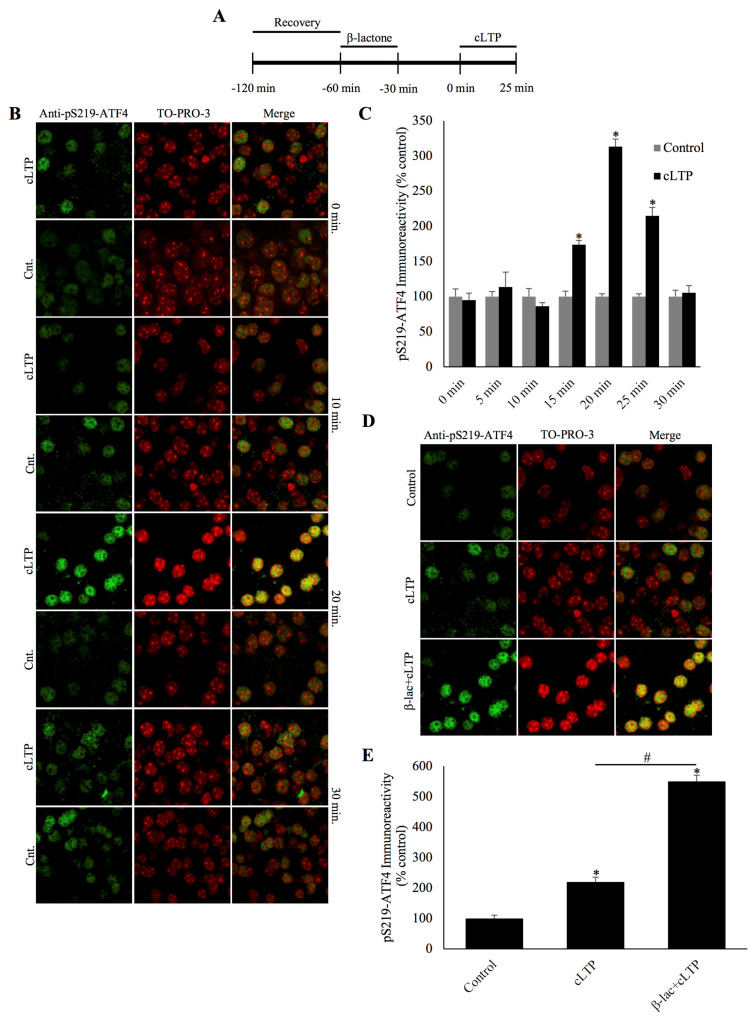
Phosphorylated activating transcription factor 4 (ATF4) is degraded during chemically induced long-term potentiation and the degradation can be blocked by a specific proteasome inhibitor β-lactone. (**A**) Schematic outline of the experiment: during the beginning of the second hour of the recovery period, the slices were treated with β-lactone for 30 min and the recovery was continued for an additional 30 min in artificial cerebrospinal fluid (ACSF). The beginning of chemically induced long-term potentiation (cLTP) induction is designated as 0 min. (**B**) Representative confocal microscopy images of phosphorylation of ATF4 at a specific serine residue (pSer219-ATF4) immunofluorescence and the nuclear counterstain TO-PRO-3. pSer219-ATF4 levels remain basal during the early timepoints after cLTP but peak at 20 min, eventually falling to levels comparable to time-matched controls by the end of the 30 min time window. (**C**) Quantification of pSer219-ATF4 immunoreactivity every 5 min during the 30 min time course, showing a peak at the 20 min time point. * *p* < 0.01 between cLTP and time-matched controls, *n* = 6, one-way ANOVA, and Tukey post hoc test. (**D**) Representative confocal microscopy images of pSer219-ATF4 immunoreactivity at 25 min after cLTP induction with treatments as indicated. pSer219-ATF4 levels are slightly higher in cLTP slices relative to time-matched controls (Cnt) and markedly increased by the pretreatment with the proteasome inhibitor β-lactone. (**E**) Quantification of pSer219-ATF4 immunoreactivity showing the significant increase caused by the inhibition of the proteasome during cLTP. * *p* < 0.01 between cLTP and time-matched controls, # *p* < 0.01 between treatment groups, *n* = 6, one-way ANOVA, and Tukey post hoc test.

It has previously been shown that after chemically-induced LTP, the quantity of total ATF4 is diminished 30 min post-stimulation and the previous experimental evidence indicated that ATF4 is degraded by the UPP [10]. In addition, the available evidence in the literature suggests that ubiquitin-proteasome-mediated degradation of ATF4 is likely to be controlled by its phosphorylation on Ser-219 [15]. Therefore, we investigated the regulation of phosphorylated ATF4 during cLTP around this time period. We collected slices that were subjected to cLTP-inducing treatments and time-matched controls every 5 min during the first 30 min time period and carried out immunohistochemical experiments using an antibody raised against ATF4 phosphorylated on Ser-219 (anti-pSer219-ATF4; Figure 1A). We then quantified phosphorylated ATF4 immunofluorescence (Figure 1A,B) and found that ATF4 phosphorylation remains low during the early part of this period (0–10 min) in both control slices and the slices subjected to cLTP but increases 15 min after cLTP induction (cLTP: 173.9% ± 5.9%, control: 99.2% ± 7.6%; *n* = 6, *p* < 0.01, one-way ANOVA, and Tukey post hoc test), with a peak at 20 min (cLTP: 313.2% ± 10.8%, control: 98.5% ± 3.9%; *n* = 6, *p* < 0.01, one-way ANOVA, and Tukey post hoc test). Phosphorylated ATF4 levels begin to fall at 25 min and reach levels comparable to controls by 30 min (Figure 1B,C; cLTP: 105.6% ± 10.0%, control: 99.3% ± 9.0%; *n* = 6, *p* = ns, one-way ANOVA, and Tukey post hoc test).

The attenuation of phosphorylated ATF4 during cLTP could be explained by its degradation by the UPP. To test this hypothesis, we incubated slices in β-lactone, a highly selective proteasome inhibitor [16]. We chose to examine the effect of proteasome inhibition at the 25 min mark as this is the timepoint between the peak and decline to normal levels of phosphorylated ATF4. As expected, phospho-ATF4 immunoreactivity was increased in the cLTP slices relative to the control slices. Furthermore, β-lactone treatment significantly increased phospho-ATF4 levels in cLTP slices (Figure 1D and 1E; β-lactone+cLTP: 550.3% ± 20.0%, cLTP: 220.0% ± 15.5%, control: 98.9% ± 10.9%; *n*=6, *p* < 0.01, one-way ANOVA, and Tukey post hoc test). These data indicate that the decline of phosphorylated ATF4 levels associated with LTP stimulation is likely to be because of proteasome-mediated degradation.

### 2.2. ATF4 Phosphorylation is Mediated by PKA during cLTP

**Figure 2:**
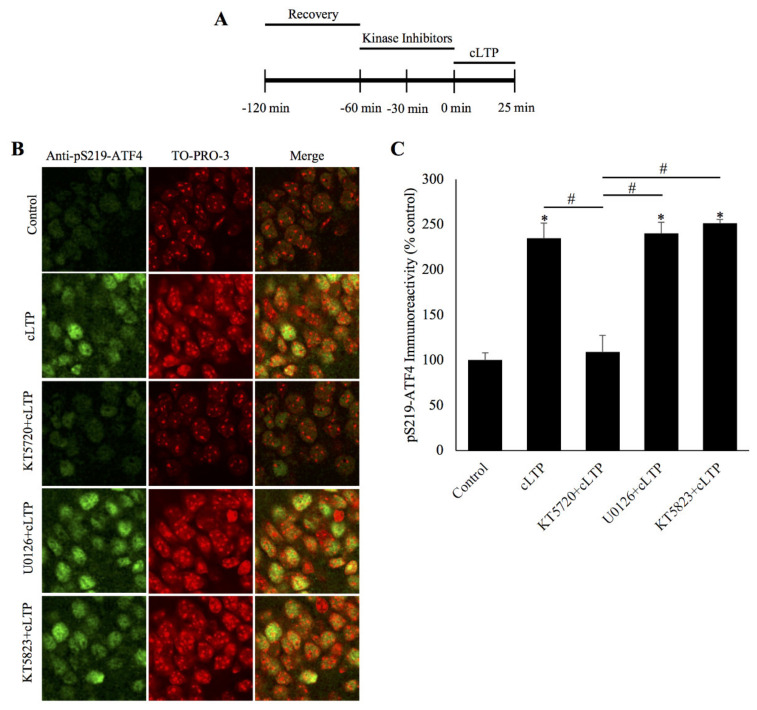
ATF4 is phosphorylated by protein kinase A during chemically induced long-term potentiation. (**A**) Schematic outline of the experiment: during the second hour of the recovery period, the slices were incubated with a kinase inhibitor. The beginning of cLTP induction is designated as 0 min. (**B**) Representative confocal microscopy images of pSer219-ATF4 and the nuclear counterstain TO-PRO-3 20 min after cLTP induction, time-matched controls, and slices pretreated with specific kinase inhibitors for either PKA (KT5720), or ERK (U0126), or PKG (KT5823) before cLTP induction. pS219-ATF4 levels are increased in cLTP slices relative to time-matched controls and this increase is significantly attenuated in slices pretreated with the PKA inhibitor KT5720 but not with the ERK inhibitor U0126 or the PKG inhibitor KT5823. (**C**) Quantification of pSer219-ATF4 immunoreactivity showing PKA inhibition prevents the phosphorylation of Ser219-ATF4 caused by cLTP at 20 min post-induction. * *p* < 0.01 between cLTP and time-matched controls, # *p* < 0.01 between treatment groups, *n* = 6, one-way ANOVA, and Tukey post hoc test.

Our data described above demonstrate that the proteasome-mediated degradation of ATF4 is at least partly dependent on the phosphorylation of Ser-219 (pSer219-ATF4). To further elucidate the mechanism of ATF4 degradation and the signaling cascade that regulates its phosphorylation, we performed a series of experiments by incubating the slices with different protein kinase inhibitors prior to cLTP induction (Figure 2A). We specifically investigated the role of three kinases that are known to play a role in long-term synaptic plasticity: cAMP-dependent protein kinase (PKA) [17], cGMP-dependent protein kinase (PKG) [18], and extracellular signal-regulated kinase (ERK) [19]. Prior incubation in KT5720 (PKA inhibitor) was found to significantly reduce ATF4 phosphorylation 20 min after cLTP induction (KT5720+cLTP: 109.3% ± 18.3%, cLTP: 234.9% ± 16.7%, control: 100.3% ± 7.9%; *n* = 6 for all groups, *p* < 0.01 for KT5720+cLTP vs. cLTP and control vs. cLTP, *p* = ns for KT5720+cLTP vs. control, one-way ANOVA, and Tukey post hoc test). However, prior incubation in the ERK inhibitor U0126 (U0126+cLTP: 240.2% ± 12.4%, cLTP: 234.9% ± 16.7%, control: 100.3% ± 7.9%; *n* = 6 for all groups, *p* < 0.01 for U0126+cLTP vs. control and control vs. cLTP, *p* = ns for U0126+cLTP vs. cLTP, one-way ANOVA, and Tukey post hoc test) or the PKG inhibitor KT5823 (KT5823+cLTP: 251.2% ± 4.3%, cLTP: 234.9% ± 16.7%, control: 100.3% ± 7.9%; *n* = 6 for all groups, *p* < 0.01 for KT5823+cLTP vs. control and control vs. cLTP, *p* = ns for KT5823+cLTP vs. cLTP, one-way ANOVA, and Tukey post hoc test) did not have a significant impact on phosphorylated ATF4 levels (Figure 2B,C). These data demonstrate that PKA is the likely kinase responsible for the phosphorylation of ATF4 during LTP. 

### 2.3. Blockade of ATF4 Degradation during cLTP by Inhibition of Neddylation Required for Activation of Skp1/Cul-1/F-box (SCF) Ubiquitin Ligases

**Figure 3:**
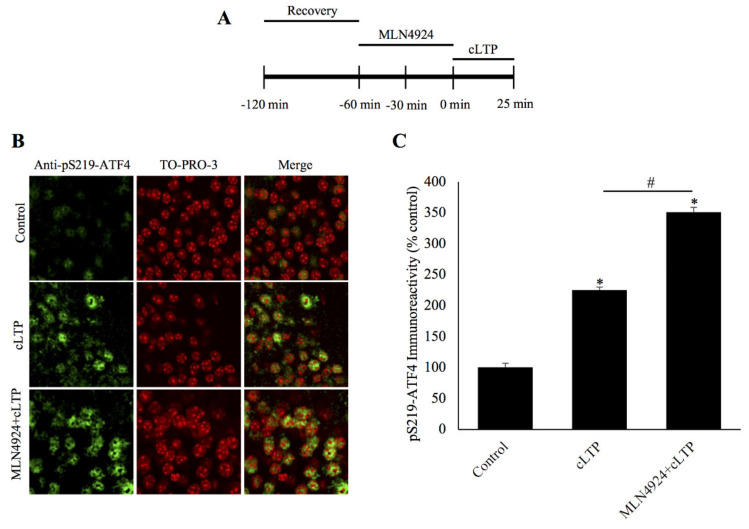
Inhibition of neddylation prevents degradation of phosphorylated ATF4 during chemically induced long-term potentiation. (**A**) Schematic outline of the experiment: during the second hour of recovery period, the slices were incubated with the neddylation inhibitor, MLN4924. The beginning of cLTP induction is designated as 0 min. (**B**) Confocal microscopy images of pSer219-ATF4 and the nuclear counterstain TO-PRO-3 25 min after cLTP induction, time-matched controls, and slices pretreated with the small molecule neddylation inhibitor MLN4924 before cLTP induction. pSer219-ATF4 levels are markedly increased relative to time-matched controls and the inhibition of neddylation significantly increases pS219-ATF4 levels after cLTP. (**C**) Quantification of pSer219-ATF4 immunoreactivity shows that the neddylation inhibitor MLN4924 causes accumulation of phosphorylated ATF4 after cLTP induction. * *p* < 0.01 between cLTP and time-matched controls, # *p* < 0.01 between treatment groups, *n* = 6, one-way ANOVA, and Tukey post hoc test.

Collectively, the data described thus far indicate that the proteasome-mediated degradation of ATF4 is correlated with formation of long-term synaptic plasticity and is likely to be regulated by its PKA-mediated phosphorylation. Polyubiquitination of ATF4 is necessary for targeting it for degradation by the proteasome. The enzyme known to be responsible for polyubiquitination of ATF4 in non-neuronal cells is an E3 ligase called SCF^βTrCP^ [15].The activation of SCF-type ligases is dependent on the neddylation of the cullin subunit. Thus, it is possible to determine whether SCF^βTrCP^ is responsible for the polyubiquitination of ATF4 by inhibiting neddylation, thereby preventing the activation of the ligase itself. To test this hypothesis, we used a small molecule inhibitor, MLN4924, which was found to specially inhibit NEDD8-activating enzyme [20] and was shown to be an effective neddylation inhibitor in neurons [21,22]. We observed that incubation in MLN4924 prior to cLTP induction resulted in a significant increase in the amount of phospho-ATF4 (Figure 3A–C; MLN4924+cLTP: 350.7% ± 8.1%, cLTP: 225.3% ± 4.5%, control: 100.6% ± 6.2%; *n* = 6 for all groups, *p* < 0.01 for MLN4924+cLTP vs. cLTP and control vs. cLTP, *p* = ns for KT5720+cLTP vs. control, one-way ANOVA, and Tukey post hoc test). These results suggest that the degradation of ATF4 is likely dependent on its ubiquitination by activated SCF^βTrCP^ E3 ligase.

## 3. Discussion

Our previous studies using immunoblots to measure the total ATF4 quantity showed that ATF4 amounts begin to decrease around 15 min after the induction of cLTP and reach their lowest levels around 30 min. The timepoint at which maximum reduction in ATF4 amounts occurred also coincided with the increase in the mRNA of *brain-derived neurotrophic factor (Bdnf)*, an immediate-early gene whose transcription is known to be driven by CREB [10]. Therefore, for the present investigation we carried out a systematic time course experiment and measured pSer219-ATF4 every 5 min from the beginning of cLTP induction. Our results revealed that phosphorylated ATF4 levels peak at 25 min post-cLTP induction and begin to return to levels comparable to controls at the 30 min timepoint.

How does the decrease in pSer219-ATF4 come about? Based on our previous results and the report that ATF4 phosphorylated on serine-19 is a substrate for UPP-mediated degradation, we tested the effect of a highly specific proteasome inhibitor β-lactone [16]. Our results showed an increase in pSer219-ATF4 rendering support to the idea that phosphorylated ATF4 is degraded by the proteasome. Although a theoretically possibility exists that a reduction pSer219-ATF4 comes about because of an increase in phosphatase activity relative to kinase activity, given the restoration of pSer219-ATF4 to maximum levels observed in our time-course studies, this possibility is not likely.

What signaling mechanisms control the degradation of ATF4 during cLTP? To answer this question, we chose to test inhibitors of three protein kinases with known roles in long-term synaptic plasticity, namely, PKA, PKG, and ERK [17,18,19]. These experiments showed a role for PKA but not for PKG or ERK in ATF4 phosphorylation. Thus, it appears that PKA regulates both the CREB activation as well as removal by its repression by ATF4.

To further elucidate the mechanism regulating the degradation of ATF4, we carried out the initial studies towards identifying the ligase responsible for ubiquitination of ATF4. Previous studies in non-neuronal cells showed that an SCF-type ubiquitin ligase containing a substrate-recognition subunit (F-Box) called β-transducin repeat containing protein (β-TrCP) is critical for ubiquitination of ATF4. These studies provided evidence that β-TrCP interacts with ATF4 phosphorylated on Ser-219 and that this particular serine reside occurs in a sequence motif DSGXXXS [15] and is important for the binding of β-TrCP to ATF4 [23]. The second serine residue (Ser-224) in the DSGXXXS motif also plays a role (albeit a minor one) in binding β-TrCP to ATF4 [23]. In the present studies, our focus has been on ATF4 phosphorylated on Ser-219. A key feature of SCF ligases is that they are enzymatically activated upon attachment of a small protein called NEDD-8 onto the cullin subunit. Neddylation of the cullin subunit is driven by the NEDD8-activating enzyme (NAE). Blockade of neddylation can be achieved by selectively inhibiting NAE with a small molecule inhibitor MLN4924 (also known as pevonedistat) [24]. We observed that the incubation of hippocampal slices with MLN4924 prior to the induction of cLTP significantly inhibited ATF4 degradation during LTP. Thus, these data provide initial evidence that the SCF ubiquitin ligase that attaches polyubiquitin to ATF4 is likely to be SCF^βTRCP^.

What is the significance of ATF4 degradation during long-term synaptic plasticity? Given that CREB-mediated gene expression is critical for long-term synaptic plasticity underlying long-term memory and that it can only go forward upon removal of ATF4-medidated inhibition, proteolytic removal of ATF4 must play a critical role. We think that ubiquitin-proteasome-mediated degradation of ATF4 is a mechanism that determines the threshold for gene expression essential for long-term synaptic plasticity that forms the basis of long-term memory.

In conclusion, our data showed that ATF4 phosphorylated on Ser-219 is a substrate for ubiquitin-proteasome mediated proteolysis during long-term synaptic plasticity and that an SCF-type ligase containing a substrate-recognition element (F-box protein) called β-TrCP is likely responsible for ubiquitination of ATF4. Our investigation lays the groundwork for understanding the molecular mechanisms that determine the threshold for long-term synaptic plasticity and memory. Considering that the perturbations of the UPP are linked to memory impairment in neurodegenerative diseases such as Alzheimer’s [25,26,27,28], ATF4 degradation by the UPP might have implications for understanding abnormalities of synaptic plasticity as well.

## 4. Materials and Methods

### 4.1. Animals

Experiments with mice (C57BL/6 male, 6–12 weeks old; Charles River, Wilmington, MA, USA) were carried out according to a protocol approved by the Institutional Animal Care and Use Committee of Georgia College & State University (Approved 11 April 2019). Animals were housed (5 animals maximum per cage) and food and water were available ad libitum. Animal husbandry was done according to the Guide for the Care and Use of Laboratory Animals (8th edition; National Academies Press, Washington, DC, USA).

### 4.2. cLTP and Treatment of Hippocampal Slices with Various Inhibitors 

Transverse hippocampal slices (400 μm) were made using a standard mechanical tissue chopper and allowed to recover in oxygenated (95% O_2_/5% CO_2_) artificial cerebrospinal fluid (ACSF) at 32 °C for 1 h. cLTP was induced using 200 nM N-methyl-d-aspartate (NMDA; Cayman Chemical, Ann Arbor, MI, USA) in 0 Mg^2+^ ACSF for 10 min followed by 0.1 μM rolipram + 50 μM forskolin (Cayman Chemical, Ann Arbor, MI, USA) in 0 Mg^2+^ ACSF for 15 min [29,30]. ACSF lacking Mg^2+^ was used as NMDA receptors are normally blocked by such ions. 

After 1 h of recovery, slices undergoing the cLTP induction protocol were incubated in ACSF with 25 μM β-lactone for 30 min or the following specific kinase inhibitors for 1 h; 20 μM U0126 (Cayman Chemical, Ann Arbor, MI, USA), 5 μM KT5720 (Cayman Chemical, Ann Arbor, MI, USA), or 5 μM KT5823 (Cayman Chemical, Ann Arbor, MI, USA); or the neddylation inhibitor MLN4924 (2 μM) (Cayman Chemical, Ann Arbor, MI, USA) followed by cLTP induction. Slices were then collected at different timepoints and processed for immunohistochemistry. 

### 4.3. Immunohistochemistry and Confocal Microscopy

After being subjected to chemically induced LTP with or without preincubation with proteasome, kinase, or neddylation inhibitors, cLTP and time-matched control slices were collected and fixed in 4% paraformaldehyde for 1 h followed by five 30 min washes with PBS at room temperature. After washing, slices were blocked in a solution containing 4% normal goat serum (Vector Laboratories), 0.4% Triton-X-100, and 0.05% sodium azide in PBS at 4 °C for 6 h. Slices were then incubated in blocking solution containing polyclonal antibody against pSer219-ATF4 (1:50; MyBioSource, San Diego, CA, USA) at 4 °C overnight. Following primary antibody incubation, slices underwent three 20 min washes in PBS containing 0.2% Triton-X-100 and were then incubated in Alexa 488-conjugated goat anti-rabbit secondary antibody (1:300; Invitrogen) and TO-PRO-3 (1:500; Invitrogen) at 4 °C for 6 h. Following secondary antibody incubation, slices underwent four 30 min washes in 0.2% Triton-X-100 in PBS and one 30 min wash in PBS. Slices were mounted with Prolong Gold antifade reagent (Invitrogen). Images were taken with an Olympus FV3000 confocal laser scanning microscope and analyzed using ImageJ (National Institutes of Health (NIH), Bethesda, MD, USA). 

### 4.4. Statistical Analysis

Data are expressed as mean ± standard error of the mean. The sample size (*n*) reflects the number of animals used for each experiment, not the number of slices. Immunoreactivity was measured in 5 slices from each animal (20 cells/slice). Data were analyzed by one-way ANOVA and Tukey post-hoc test.
